# Efficacy and safety of praziquantel against *Schistosoma haematobium* in the Ikata-Likoko area of southwest Cameroon

**DOI:** 10.1186/s41182-017-0071-8

**Published:** 2017-12-18

**Authors:** Calvin Bisong Ebai, Helen Kuokuo Kimbi, Irene Ule Ngole Sumbele, Jude Ebah Yunga, Leopold Gustave Lehman

**Affiliations:** 1 Department of Zoology and Animal Physiology, Faculty of Science, University of Buea, P.O. Box 63, Buea, SWR Cameroon; 2 Department of Medical Laboratory Sciences, Faculty of Health Sciences, University of Bamenda, P O Box 39, Bambili, NWR Cameroon; 3 Department of Animal Biology, Faculty of Science, University of Douala, P.O. Box 24157, Douala, Cameroon

**Keywords:** Cameroon, Efficacy, Egg reduction rate, Praziquantel, Safety, *Schistosoma haematobium*

## Abstract

**Background:**

Schistosomiasis remains a parasitic infection of public health importance especially in Africa south of the Sahara including Cameroon. Chemotherapy using praziquantel has been the most effective and widespread control measure used. However, there are reports of reduced efficacy of the drug. The aim of this study was to assess the efficacy and safety of praziquantel against *Schistosoma haematobium* among infected individuals in the Ikata-Likoko area of southwest Cameroon. Following a baseline study, *S. haematobium* egg load was determined using the urine filtration technique and microscopy. Participants were treated with a unique dose of praziquantel of 40 mg/Kg body weight. A control test was carried out on the 42nd day post-treatment to determine the proportion of positive participants with viable eggs (cure rate) and the egg loads. The egg loads obtained during the control and at baseline were used to calculate the egg reduction rate (ERR) used as the main indicator of praziquantel efficacy according to the WHO, 2013 protocol.

**Results:**

At baseline, the prevalence of *S. haematobium* was 34.3% (177/516). Out of these a total of 174 participants aged between 4 and 76 years were recruited into the study. A total of 130 participants came for follow up on day 42. Among them, 22.3% (29) were positive for eggs of *S. haematobium* but none of the eggs were viable giving a cure rate of 100%. The overall mean egg load per 10 mL (MEL/10 mL) of urine reduced from 31 (1–400) at baseline to 6.0 (1–35) on day 42. The overall ERR was reduced (80.3%). However, the efficacy was satisfactory (ERR ≥ 90%) in females, children ˂ 5 years, and some localities and for individuals with heavy infection intensity. Fifteen (8.6%) of the participants presented minor adverse events including abdominal disorders, headache and vomiting but did not last for more than 24 h.

**Conclusions:**

Treatment with praziquantel was efficacious and safe showing reduction in prevalence as well as mean egg load in some individuals with few adverse events recorded. The distribution of praziquantel in the area should be extended to other age groups and not just school-age children. A study with multiple drug doses and longer period of evaluation could reveal more information on praziquantel efficacy in the area.

## Background

Schistosomiasis remains a parasitic infection of public health importance in many tropical and subtropical countries especially in Africa south of the Sahara. The World Health Organization (WHO) estimates that up to 218.8 million people in the world with a majority of them in sub-Saharan Africa required treatment for schistosomiasis in 2015 [1]. Effectively, more than 66 million received preventive chemotherapy with praziquantel (PZQ). In addition to chemotherapy, other measures to fight against the disease include snail (intermediate host) control, basic sanitation, supply of safe water and health education either separately or in combination [2]. Globally, chemotherapy has been the most widespread antischistosomal measure used. Although, several drugs have been found to be efficacious against all five species of *Schistosoma* [3], praziquantel has been the most widely used. It is a pyrazinoquinoline derivative whose safety and efficacy have ensured its widespread usage. Although it is found to be associated with minor adverse events such as abdominal disorder, nausea and vomiting [4, 5], PZQ remains active against adult schistosomes [6]. It has also been reported to improve on the morbidity of the disease, with some clinical signs and symptoms such as haematuria, abdominal pain and dysuria subsiding shortly following treatment [7–9]. Considering that the disease has a focal distribution, the WHO recommends that distribution of praziquantel should be done following the prevalence in the communities. In this regard, in high-risk communities where parasitological prevalence is ≥ 50% and visible haematuria is ≥ 30%, children of school age and adults considered to be at risk should be treated once a year. Meanwhile, in moderate-risk communities where parasite prevalence is ≥ 10% but ˂ 50%, all school-age children and adults considered to be at risk should receive treatment once every 2 years. In the same light, in low-risk communities where parasite prevalence is ˂ 10%, all school-age children should be treated twice during primary school age while praziquantel is made available in health care institutions for treatment of suspected cases. Unfortunately, reports have indicated the availability of suboptimal brands of the drug in the market thus adding to the already existing pressure on the drug and to the reported resistance developed by the parasites [10, 11].

Urinary schistosomiasis caused by *Schistosoma haematobium* is reportedly prevalent in Cameroon [12–16], and its main control measure has been mass distribution of PZQ to school children since 2004. Unfortunately, there have been reports about the increasing problem of reduced efficacy of the drug in some countries [17] such as Zimbabwe [18], Egypt [19] and Cameroon [10, 15]. However, there has been no assessment of its efficacy in many foci in the Southwest Region of Cameroon including the Ikata-Likoko area in the Mount Cameroon Region where the disease is prevalent [14, 16]. Against this background, this study was carried out to assess the efficacy and safety of PZQ in the Ikata-Likoko area of southwest Cameroon.

## Methods

### Study area

This study was carried out in the Ikata-Likoko area comprised of four rural localities, Ikata, Bafia, Mile 14 and Likoko in the southwest of Cameroon. Ikata is located between longitudes 9.363 E and 9.352 E and latitudes 4.329 N and 4.328 N and between 87 and 132 m above sea level. Bafia is between longitudes 9.324 E and 9.311 E and latitudes 4.350 N and 4.363 N and is 229 to 256 m above sea level. Mile 14 is located between longitudes 9.302 E and 9.292 E and latitudes 4.396 N and 4.401 N and between 157 and 168 m above sea level. Likoko is located between longitudes 9.319 E and 9.320 E and latitudes 4.399 N and 4.393 N and is between 108 and 116 m above sea level. Access to these villages from the main town is through an earth road which is usually muddy in the rainy season. The topography of the area is characterized by hills and valleys. Rainfall averages 3126.7 mm annually while temperature varies between 23 and 33 °C with an annual average of about 26.2 °C. There are two major seasons in the area, the rainy (March to October) and the dry (November to February) seasons. The vegetation is mainly the tropical forest type. More details on the study area were already published with baseline data of this study by Ebai et al. [20]. Earlier studies by Ntonifor et al. [14] and Kimbi et al. [16] carried out in localities near this study area have shown that only *S. haematobium* is present in the area. Mass distribution of praziquantel by the Ministry of Health in Cameroon is done in schools. Personal communication with village authorities and participants indicate that the last distribution of praziquantel in the schools dated more than 2 years before this study was started.

### Study population

Participants in this study were individuals who had spent at least 2 months in the study area, were aged 1 year and above, were positive for *S. haematobium* in the baseline study and who gave their informed consent. Assent for minors was obtained from parents or legal guardians.

Participants with signs of chronic or severe illness, such as cardiac, renal or hepatic disease, HIV/AIDS, severe diarrhoea, and dehydration, and those with history of treatment with PZQ in the past 2 months, with hypersensitive reaction to PZQ, and pregnant or breast feeding mothers were excluded from the study [3]. Also, participants with residence out of the study area and those who violated the study protocol, used other antischistosomal drug, and withdrew their assent or consent or could not be attended to during follow up were excluded from the study.

### Study design

This was a prospective study carried out between June and September, 2014. After obtaining administrative and ethical clearances, visits to the village authorities were scheduled during which the procedures and the benefits of the study as well as the dates and collection venues for the study were presented. A structured questionnaire was used to collect data on the socio-demography of the participants. Urine samples were collected and transported to the Malaria Research Laboratory of the University of Buea for parasitological analyses. Individuals who were positive for *S. haematobium* were treated with praziquantel and enrolled into the study. The follow up was done on the 42nd day post-treatment, during which parasitological analyses were repeated to detect the presence, density and viability of *S. haematobium* eggs.

### Administration of questionnaire

Socio-demographic data collected through the use of questionnaire included age, sex, level of educational attainment, religion and occupation. The body weight was measured using a floor scale (Seca GmbH & Co. Germany). Concerning general health, participants were asked if they were suffering from any chronic disease, were on any treatment or had taken PZQ within the last 6 months. Questionnaires were administered in English and exceptionally in Pidgin English where necessary.

### Urine sample collection and laboratory analyses

Labelled 50-mL containers were given to participants for the collection of urine samples. Collection was done between 10 a.m. and 2 p.m. which is the period corresponding with the peak excretion of schistosome eggs [21]. Immediately after collection, the samples were tested biochemically for the presence of blood (haematuria) using urine test strips (Medi test Combi 9, Germany). The urine containers were corked and transported in cool boxes to the Malaria Research Laboratory of the University of Buea for parasitological analyses.

Ten milliliter of each urine sample was analysed for schistosome eggs using the syringe filtration method as described by Cheesbrough [21]. Filtration was done by passing 10 mL of urine through a filter (STERLITECH Corporation, USA) which retains the schistosome eggs. The filter was placed on a microscope slide and examined under × 10 objective of a light microscope (Olympus, USA). The number of eggs counted was reported per 10 mL of urine. Eggs detected were tested for viability by adding a drop of methylene blue on every positive slide. Viable eggs remained colourless while non-viable eggs were stained blue [21]. Participants who had ≤ 50 eggs/10 mL of urine were classified as light infections while those with ˃ 50 eggs/10 mL of urine were heavy infections.

### Treatment and follow up of *S. haematobium* infection

Participants who were positive for eggs of *S. haematobium* were treated with praziquantel oral tablets (Cesol™ 600, Germany, batch number M25343) at 40 mg/Kg body weight in a unique dose alongside snacks as recommended by WHO [3]. Those who received the tablets were under observation for at least 4 h, and any adverse events were recorded. Adverse events were defined as any manifestation that was absent at baseline but present after swallowing the praziquantel tablets. On day 42 post-treatment, urine samples were collected from the participants and analysed for *S. haematobium* eggs. When found, the eggs were counted and tested for viability. Participants who did not show up for the control were contacted on telephone and attended to in their homes. Those who were not attended to on this day were excluded from the study. This was to avoid reinfection cases.

### Endpoints for follow up of praziquantel efficacy

The primary endpoint of this study was the efficacy of PZQ which was classified into three levels by comparing the observed egg reduction rate (ERR) with the 90% reference value [3]. Hence, the drug efficacy was either of the following: satisfactory if the ERR ≥ 90%, reduced if the ERR ≥ 80% but < 90% or doubtful if the ERR < 80%. The secondary endpoints were the prevalence at follow up and the cure rate. A cured participant in this study was defined as one who was positive for urinary schistosomiasis (US) with viable eggs on day 0, received PZQ treatment and was negative for US on day 42, or was positive for US but the eggs detected were non-viable [3].

### Statistical analysis

Data was entered into Excel version 2013 and analysed using IBM Statistical Package for Social Sciences (IBM SPSS) version 20 (IBM Inc. 2012). Results were summarized into proportions and means. Proportions were compared using the Cramer’s (*V*) and chi-square (*χ* ^2^) tests, and the Mann-Whitney test (*U*) was used to compare mean egg loads for two groups while the Kruskall-Wallis test (*H*) was used to compare mean egg loads for more than two groups. The Wilcoxon signed-rank test (*Z*) was used to compare mean egg loads at day 0 and day 42. The ERR was calculated from the formula: ERR (%) = 100 × 1 − [arithmetic mean egg counts on day 42/arithmetic mean egg counts at baseline] [3]. The level of significance was set at *P* < 0.05.

## Results

### Characteristics of the study population

At baseline, the prevalence of *S. haematobium* was 34.3% (177/516). Out of the 177 participants who were positive for *S. haematobium*, a total of 174, 84 males and 90 females, with an age range of 4–76 years and mean age ± SD of 23.8 ± 17.5 years were enrolled into the study (Table 1). They had a MEL/10 mL of urine of 30 (1–400). All the eggs detected were viable. None of the participants had taken antischistosomal treatment during the past 6 months before the commencement of the study. Out of the 174 participants enrolled, 130 (74.7%) came for follow up on day 42 out of which 29 (22.3%) were positive for US with all the eggs being non-viable, giving a cure rate of 100%. On day 42 post-treatment, the overall MEL/10 mL of urine was 6.0 (range, 1–35). The overall efficacy was reduced with an egg reduction rate (ERR) of 80.3%.

**Table 1:** Characteristics of participants in the monitoring of praziquantel efficacy on *S. haematobium* in the Ikata-Likoko area of southwest Cameroon (*n* = 174)

Characteristics	Category	Frequency/value	Percentage (%)
Sex	Male	84	48.3
	Female	90	51.7
Age group (years)	< 5	23	13.2
	5–15	54	31
	> 15	97	55.8
Mean age ± SD (years)		23.8 ± 17.5	
Prevalence of *S. haematobium*	Day 0	177/516	34.3
Number admitted into the study	Day 0	174	
Overall mean egg load/ 10 mL of urine	Day 0	30 (range, 1–400)	
Highest level of school attainment	No formal education and primary	130	74.7
	Secondary and tertiary	44	25.3
Occupation	Semi-skilled workers	7	4.0
	Farmers	66	38.0
	Housewife	6	3.4
	Pupils and students	95	54.6

### Efficacy of praziquantel with respect to gender and age

With respect to gender, the prevalence of US reduced significantly in both sexes following treatment, from 100% on day 0 to 10.0% on day 42 for the male participants and from 100 to 12.3% for the females. Comparing the prevalence and mean egg load after treatment for males and females did not show any statistically significant difference (Table 2). On the contrary, there were significant reductions in MEL/10 mL of urine from 32 to 12 for the males (*Z* = 3.854, *P* < 0.001) and from 29 to 3 for the females (*Z* = 4.231, *P* < 0.001). The ERR was satisfactory in females (90%) but doubtful in males (62.5%).

**Table 2:** Efficacy of praziquantel against *S. haematobium* with respect to gender and age in the Ikata-Likoko area of southwest Cameroon

Characteristic	Category	Prevalence on day 0 (*n*) (*N* = 174)	Prevalence on day 42 (*n*) (*N* = 130)	Mean egg load on day 0 (range)	Mean egg load on day 42 (range)	*Z* test *P* value	ERR (%)
Sex	Male	100 (84)	10.0 (13)	32 (1–400)	12 (2–35)	3.854 < 0.001	62.5
	Female	100 (90)	12.3 (16)	29 (1–300)	3 (1–10)	4.231 < 0.001	90.0
Level of significance		*χ* ^2^ = 0.06, *P* = 0.80		*U* = 1854.5, *P* = 0.109			
Age	< 5	100 (23)	0.0 (0)	38 (1–300)	0.0	2.805 0.005	100
	5–15	100 (54)	9.2 (12)	36 (1–200)	6 (1–35)	3.626 < 0.001	83.3
	> 15	100 (97)	13.1 (17)	24 (1–400)	6 (1–35)	3.847 < 0.001	75.0
Level of significance		*χ* ^2^ = 15.55, *P* = 0.0004		*H* = 2.03, *P* = 0.02			

There were significant reductions in the prevalence of US in all age groups (*P* = 0.0004) following treatment as well as the MEL/10 mL (*P* = 0.02). The ERR was highest in children less than 5 years (100%) when compared with other age groups.

### Efficacy of praziquantel with respect to socio-demographic factors

In terms of occupation, a statistically significant reduction in prevalence (*V* = 0.2, *P* = 0.0007) was observed with the highest reduction recorded among farmers, from 100% (66) on day 0 to 7.0% (9) on day 42 while, the least reduction was among semi-skilled workers, from a prevalence of 100% (7) to 1.5% (2). Although there was a reduction in the mean egg loads/10 mL of urine among the different occupations, the difference was not statistically significant (*H* = 3.5, *P* = 0.32) (Table 3).

**Table 3:** Efficacy of praziquantel against *S. haematobium* with respect to socio-demographic factors and locality

Characteristic	Category	Prevalence at day 0 (*n*) (*N* = 174)	Prevalence at day 42 (*n*) (*N* = 130)	Mean egg load on day 0 (range)	Mean egg load on day 42 (range)	*Z* test *P* value	ERR (%)
Occupation	Semi-skilled worker	100 (7)	1.5 (2)	15 (4–50)	3 (1–5)	1.604 0.109	80.0
	Farmers	100 (66)	7.0 (9)	25 (1–400)	7 (1–35)	1.605 0.108	72.0
	Housewife	100 (6)	1.5 (2)	30 (5–50)	5 (1–10)	2.821 0.005	83.3
	Pupil/student	100 (95)	12.3 (16)	34 (1–300)	6 (1–35)	1.342 0.180	82.4
Level of significance		*V* = 0.20, *P* = 0.0007		*H* = 3.5 *P* = 0.32			
Locality	Bafia	100 (20)	4.6 (6)	53 (1–400)	5 (1–10)	1.572 0.116	90.6
	Ikata	100 (33)	5.4 (7)	48 (2–300)	3 (1–6)	2.154 0.012	93.8
	Likoko	100 (48)	2.3 (3)	20 (1–200)	7 (2–35)	3.117 0.002	65.0
	Mile 14	100 (73)	10.0 (13)	23 (1–50)	9 (1–35)	3.923 < 0.001	74.3
Level of significance		*V* = 0.163, *P* = 0.242		*H* = 6.1, *P* = 0.12			
Highest level of school attainment	No formal or primary	100 (130)	16.1 (21)	31 (1–400)	5 (1–35)	4.919 < 0.001	83.8
	Secondary/tertiary	100 (44)	6.0 (8)	30 (1–200)	8 (1–35)	1.965 0.049	73.3
Level of significance		*χ* ^2^ = 4.25, *P* = 0.04		*U* = 1730, *P* = 0.61			

A significant reduction in prevalence post-treatment was observed in all the localities, with the highest reduction occurring in Mile 14 from 100% (73) to 10% (13), whereas the least prevalence was recorded in Likoko, from 100% (48) to 2.3% (3). MEL/10 mL of urine reduced significantly in three of the localities (*P* ˂ 0.05) except in Bafia (*Z* = − 1.572, *P* = 0.116). Satisfactory ERR were observed in Bafia (90.6%) and Ikata (93.8%), whereas those of Likoko (65%) and Mile 14 (74.3%) were doubtful.

The prevalence of US reduced significantly (*χ* ^2^ = 4.25, *P* = 0.04) by day 42 in both groups of levels of educational attainment. Among participants with no formal or primary education, the prevalence reduced from 100% (130) to 16.1% (21) after treatment while that for participants who had attained secondary or tertiary education reduced from 100% (44) to 6.0% (8). No significant differences in MEL/10 mL of urine were observed on day 0 and day 42, with respect to level of education (*U* = 1730, *P* = 0.61). On the other hand, the ERR was higher in participants with no formal or primary education (83.8%) than their counterparts with secondary or tertiary education (73.3%).

### Efficacy of praziquantel with respect to initial infection intensity

With respect to infection intensity, the difference in US prevalence between day 0 and day 42 was statistically significant between participants with initial heavy infections (1.6%) and those with light infections (20.8%) down from 100% on day 0 (*V* = 0.3, *P* ˂ 0.0001). A statistically significant difference was observed in MEL/10 mL of urine in both categories (Table 4). For the light infections, the MEL/10 mL of urine reduced from 15.53 to 6.15 (*Z* = 5.33, *P* ˂ 0.001) while for the heavy infections, it reduced from 153.58 to 6.0 (*X* = 2.03, *P* = 0.04). The ERR was satisfactory and higher in individuals with heavy infections (96.1%) than those with light infections (60.4%).

**Table 4:** Efficacy of praziquantel against *S. haematobium* on day 42 post-treatment with respect to initial infection intensity in the Ikata-Likoko area of southwest Cameroon

Characteristic	Category	Prevalence day 0 (*n*) (*N* = 174)	Prevalence day 42 (*n*) (*N* = 130)	Mean egg load on day 0 (range)	Mean egg load on day 42 (range)	*Z* test *P* value	ERR (%)
Infection intensity	Light ≤ 50 eggs/10 mL	100 (155)	20.8 (27)	15.53 (1–50)	6.15(1–35)	5.33 < 0.001	60.4
	Heavy > 50 eggs/10 mL	100 (19)	1.6 (2)	153.58 (56–400)	6.00(4–8)	2.03 0.04	96.1
	Level of significance	*V* = 0.3, *P* < 0.0001		*U* = 4.32, *P* = 0.13			
Overall		100 (174)	22.3(29)	31 (1–400)	6 (1–35)	5.73 < 0.001	80.3

Also, it was observed that before treatment, 60 (34.5%) participants who were positive for US presented with haematuria, but after treatment, all of them were negative for haematuria.

**Fig. 1:**
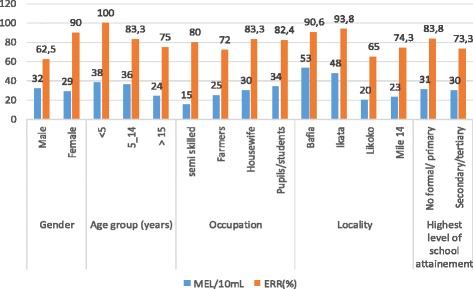
Variation in ERR on day 42 post praziquantel treatment with mean egg loads per category on day 0

With respect to mean egg loads in the different categories, it was also observed that a majority of participants with higher mean egg load at admission had higher ERRs. In the age category, children below 5 years who presented with the highest initial egg load had the highest ERR of 100%. Similarly, in terms of occupation, housewives and pupils/students who had relatively lower initial MEL/10 mL of urine of 30 and 34, respectively, had ERR of 83.3 and 82.5%. These were higher than the values observed in farmers and semi-skilled participants with lower initial egg loads. In like manner, participants of Bafia and Ikata with higher initial MEL/10 mL of urine of 53 and 48, respectively, had higher ERR of 90.6 and 93.8% than those of Mile 14 and Likoko with lesser initial MEL/10 mL of urine as shown on Fig. 1.

### Adverse events post-treatment with praziquantel

Some participants (15, 8.6%) amongst the 174 recruited manifested adverse events such as abdominal pain (6, 3.4%), nausea (7, 4.0%) and headache (6, 3.4%) hours after treatment with praziquantel. The prevalence of adverse events amongst participants was comparable. Two (1.1%) participants had all three adverse events. Nonetheless, these events did not last for more than a day in all the participants.

## Discussion

Treatment with praziquantel is the mainstay for the control of schistosomiasis in endemic areas. However, with reports on decreased cure rates in some endemic areas including Cameroon [15, 17, 18], there is a need for continuous monitoring of its efficacy while continuing to seek a possible replacement when the need arises.

The results obtained from this study indicate that a single dose of PZQ (40 mg/kg body weight) had a reduced efficacy (ERR, 80.3%) 42 days post-treatment with respect to the 90% efficacy recommended by WHO protocol [3]. The efficacy observed in this study is less than that recorded by Tchuem-Tchuente et al. in the Littoral Region of Cameroon [10]. It is however similar to that recorded by Tchuem-Tchuente et al. in the Northern and Central Regions of Cameroon [15] and Ojurongbe et al. in Nigeria [8]. This observed efficacy could be due to reasons including the fact that follow up was done once, for a shorter period, and that only a unique dose of the drug was administered. Similar studies with multiple doses of treatment and longer periods of follow up [8, 10] have reported higher efficacies. Longer periods of follow up may depict constant reduction in egg loads with time. Given that schistosomiasis is generally chronic, egg release in most cases starts way before treatment is sought especially in areas where praziquantel is not readily available. Some eggs may be trapped in tissues and some probably excreted after treatment. Egg excretion in a majority of cases continues months after the death of the worms [22]. The late excretion of eggs results in a reduction in the observed efficacy of the drug given that the mean egg load on the follow-up day is increased.

Variation in efficacy was noted with some factors such as sex, age and locality, where the females, children < 5 years and those from Bafia as well as Ikata showed satisfactory efficacies. This variation could be due to factors associated with individuals, such as general health condition, cognisant of the fact that not all health conditions were excluded in this study. Also, it could be due to parasite loads which varied in the different categories of participants or prevailing environmental factors. These findings are similar to those obtained by Senghor et al. in Senegal [23]. However, the higher efficacy observed in females could be buttressed by the fact that female hormones increase antibody response to specific parasites resulting in a higher female resistance against several parasitic infections [24, 25]. Also, females may have better complied to advice given them during the study knowing that they are more exposed to infection as their daily domestic activities bring them in regular contact with infested water bodies. Similarly, following treatment, the prevalence and egg loads of *S. haematobium* reduced in the study population, an indication that the adult schistosomes were effectively killed by the drug. The reduction in prevalence and egg load varied within some of the categories including age, occupation, level of education and initial infection intensity. Further studies on the influence of these factors on the bioavailability and pharmacokinetics of the praziquantel may provide more information on the efficacy of the medication.

With respect to infection intensity, the higher ERRs observed in participants with higher initial infection intensity probably suggests a higher efficacy of praziquantel on heavy intensity infections than light ones. These results are contrary to the findings of Ojurongbe et al. in Abeokuta, Nigeria [8] and Tchuem-Tchuente et al. [15] in Cameroon. In addition, factors such as high pre-treatment egg intensities, poor drug absorption and a high rate of PZQ catabolism, rather than parasite resistance, have been attributed to reduced PZQ cure rate in some endemic areas [18].

The decrease in prevalence of haematuria among participants after treatment is a confirmation of the efficacy of praziquantel in the reduction of morbidity associated with US as reported by earlier studies [8, 26]. The elimination of the worms by the drug would lead to a corresponding decrease in the number of eggs laid. Eventually, the egg numbers that traverse blood vessels in the bladder reduce and so the amount of blood lost as haematuria is reduced.

## Conclusion

Based on ERR rate, the efficacy of praziquantel in the Ikata-Likoko area was reduced although it varied from doubtful to satisfactory with respect to socio-demographic factors. On the other hand, the efficacy in terms of cure rate was 100% since no viable eggs were detected among all positive individuals during control on day 42. Treatment with PZQ showed variation in the reduction of prevalence between age groups, occupations, level of education, initial infection intensity and variation in the mean egg load of the different age groups. Praziquantel can be described as safe due to the few minor adverse events observed. Efficacy is also confirmed since a significant decrease in haematuria was observed after treatment. The distribution of PZQ in the area should be extended to other age groups and not just to school children. Another study with a prolonged period of assessment could improve the evaluation of efficacy of PZQ on urinary schistosomiasis in the study area.

## Abbreviations

ERR: Egg reduction rate
*H*: Kruskall-Wallis test
IBM SPSS: IBM Statistical Package for Social Sciences
MEL/10 mL: Mean egg load per 10 mL
PZQ: Praziquantel
*U*: Mann-Whitney test
US: Urinary schistosomiasis
WHO: World Health Organization
*χ*^2^: Chi-square test
*Z*: Wilcoxon signed-rank test
